# Iodine and Carbolic Acid

**Published:** 1868-06

**Authors:** 


IODINE AND CARBOLIC ACID.—The *Journal des Connaissances Medicales* publishes a letter addressed to Dr. Caffe on Dr. Percy Boulton's late discovery of the action of carbolic acid on iodine. "the inconvenience," says the writer, attending the external application of iodine and its preparations, is so serious that physicians are often compelled to abandon a remedy the therapeutic efficacy of which is undoubted, nay, almost unequalled *materia medica.* The great objection to the external use of this remedy is, that it leaves marks both on the linen and on the skin. This is a sufficient motive for seeking some means of getting rid of this drawback, especially in the case of ladies. Dr. Percy Boulton's method consists in adding a few drops of phenic (carbolic) acid to the iodine solution to be employed. This addition renders iodine perfectly colorless, so that it may be applied with impunity. But this combination has another advantage. It appears from that practitioner's observations, which I can confirm, that, so administered, carbolate of iodine, which is the new substance in question, is not only one of the most powerful antiseptics we possess, but is intrinsically a more efficacious agent than iodine alone. I have used this compound under the form of injections, gargles, and lotions, in all cases in which iodine is prescribed. In sore throat, ozæna, abscess in the ear, etc., this preparation is a sovereign remedy; since, besides its disinfecting qualities, it modifies the mucous membrane, causes all local sensibility to disappear, and cures the patient much sooner than if either of the two agents were employed separately. The formula I employ is as follows: Compound tincture of iodide, 3 gms.; pure liquid carbolic acid, 6 drops; glycerine, 30 gms.; distilled water, 150 gms.

